# Improving Management Performance of P2PSIP for Mobile Sensing in Wireless Overlays

**DOI:** 10.3390/s131115364

**Published:** 2013-11-08

**Authors:** Pablo Sendín-Raña, Francisco Javier González-Castaño, Felipe Gómez-Cuba, Rafael Asorey-Cacheda, José María Pousada-Carballo

**Affiliations:** 1 Centro Universitario de la Defensa, University of Vigo, Praza de España, s/n, 36920 Marín, Spain; E-Mails: pablo@cud.uvigo.es (P.S.-R.); chema@cud.uvigo.es (J.M.P.-C.); 2 AtlantTIC, University of Vigo, Campus s/n, 36310 Vigo, Spain; E-Mail: fgomez@gti.uvigo.es; 3 Gradiant, Campus s/n, 36310 Vigo, Spain; E-Mail: javier@det.uvigo.es

**Keywords:** P2P overlays, DHT, P2PSIP

## Abstract

Future wireless communications are heading towards an all-Internet Protocol (all-IP) design, and will rely on the Session Initiation Protocol (SIP) to manage services, such as voice over IP (VoIP). The centralized architecture of traditional SIP has numerous disadvantages for mobile *ad hoc* services that may be possibly overcome by advanced peer-to-peer (P2P) technologies initially developed for the Internet. In the context of mobile sensing, P2PSIP protocols facilitate decentralized and fast communications with sensor-enabled terminals. Nevertheless, in order to make P2PSIP protocols feasible in mobile sensing networks, it is necessary to minimize overhead transmissions for signaling purposes, which reduces the battery lifetime. In this paper, we present a solution to improve the management of wireless overlay networks by defining an adaptive algorithm for the calculation of refresh time. The main advantage of the proposed algorithm is that it takes into account new parameters, such as the delay between nodes, and provides satisfactory performance and reliability levels at a much lower management overhead than previous approaches. The proposed solution can be applied to many structured P2P overlays or P2PSIP protocols. We evaluate it with Kademlia-based distributed hash tables (DHT) and dSIP

## 1. Introduction

Wireless communications are heading towards an all-Internet Protocol (all-IP) approach, in which phone services provided by IP multimedia subsystems (IMS) will rely on the Session Initiation Protocol (SIP) for management purposes. However, traditional SIP has a centralized architecture, and therefore, peer-to-peer (P2P) networking may improve its resilience in wireless environments.

SIP is a signaling protocol commonly used for network management and data exchange applications, such as voice over IP (VoIP) or sensor information. Among other features, SIP provides high scalability and flexibility. These are desirable features in heterogeneous mobile networks of arbitrary size [1] in which people no longer only communicate in the traditional way, but also share other information that is relevant to the *context* of their activities [2]. In fact, nowadays, many phone services rely on context-aware information provided by *embedded sensors*, which the users share. This imposes the need to achieve fast and seamless data delivery between network devices, for which SIP offers reliable support. In the context of mobile sensing, P2PSIP protocols facilitate decentralized and fast communications between sensor-enabled terminals. Nevertheless, in order to make P2PSIP protocols feasible in mobile sensing networks, it is necessary to minimize overhead transmissions for management purposes, thereby reducing battery lifetime.

P2P services are often based on overlay networks, in which resource management is a key challenge. P2P network states are strongly transient, and routing survivability to failures is a very important design consideration [3]. In practice, churn (peers leaving or joining the network) can affect the accuracy of the routing and resource tables at the peers, because certain entries (corresponding to individual peers) may be missing or stale. This affects both the efficiency and consistency of operations, such as lookups. While incomplete distributed hash tables (DHT) degrade performance (the less complete the registration information, the more hops the lookup procedure needs), stale contacts increase latency, due to timeouts (when peers that have waited a defined time for a response decide that the requested peer no longer belongs to the overlay). Since timeout intervals typically last no longer than a few round-trip times, latency can easily exceed the needs for common real-time operations. This is a greater problem in mobile wireless networks, where topology is inherently more unstable than in the wired Internet.

Peers that participate in a P2PSIP [4] overlay not only act as conventional SIP user agents (UAs), but also, collectively, play the normal roles of a central server. In such a system, registrar servers (where registration information is placed), proxy servers (intermediary nodes) and message routing functions of the traditional SIP protocol are replaced by a distributed P2P overlay. The core functions of this overlay are storage, discovery and access to digital resources. Currently, the P2PSIP working group of the Internet Engineering Task Force (IETF) is considering proposals for the P2PSIP peer protocol based on distributed SIP (dSIP) [4] and Kademlia [5]. dSIP is an SIP-based protocol that has been proposed as a generic framework for a distributed SIP location service. In other words, it is a protocol for resource lookup in peer-to-peer SIP networks. It is simple and reusable with traditional SIP UAs. We describe Kademlia in Section 2.3.

P2PSIP with DHT is a realistic alternative for large decentralized deployments [6]. According to a study of three different evolution paths for mobile peer-to-peer (MP2P) communications in [7], it is a strong candidate for Internet VoIP support in worldwide interoperability for microwave access (WiMAX) or long term evolution (LTE) networks. Kademlia and dSIP are suitable for P2PSIP implementation on wireless networks. In this context, they yield good routing performance and scalability, allow self-organization and achieve secured, trustworthy P2P overlays. In [8,9], it is demonstrated that a Kademlia-based implementation can support general Internet services and distributed multimedia services on wireless networks. Kademlia's exclusive-OR (XOR) topology-based routing schema requires less maintenance than other structured P2P overlays [10], such as Pastry [11] or Tapestry [12]. It also performs better than Chord [13] in terms of lookup routing, overlay routing scalability and overlay self-organization.

From a practical perspective, we chose Kademlia for our study, because there is a real implementation of dSIP + Kademlia available (from the University of Parma [14]); the dSIP implementation is fully compatible with current commercial SIP solutions, and it is relatively simple to modify without compromising compatibility.

Some goals of P2PSIP systems are:

- Automatic setup: neighbor discovery or initial registration should be automatic procedures.
- Efficient lookup: the system should scale well with an increasing number of peers and growing demands.
- Support for heterogeneous peers: the participants in the overlay should be able to own different resources and have different network and available capacities; the system must also be platform-independent.
- Interoperability: A P2PSIP and another SIP-compliant node (even with traditional SIP implementations) should understand each other.

In order to keep the DHT registers of the P2PSIP network up-to-date, they are refreshed periodically. This paper discusses previous algorithms for managing *time-to-refresh (TTR)* timers and proposes an adaptive algorithm to optimize these timers to ensure the consistency of distributed routing tables and resource registers in P2P wireless overlay networks. This goal is important, since it will improve the trade-off between management cost and updating accuracy. With static *TTR* algorithms, management load grows with updating accuracy. The proposal, called adaptive *TTR* (*ATTR*), outperforms previous approaches in terms of management signaling overhead, by taking into account latencies between wireless network peers and by setting up a separate *TTR* timer for each P2PSIP resource to be refreshed, rather than a common timer per peer. Our work focuses on structured overlay networks [15], such as Kademlia [16] (employed by the eDonkey P2P system [17,18]) and Chord [13]. As previously mentioned, a joint Kademlia-based DHT/dSIP implementation of P2PSIP was considered. Following the Kademlia philosophy of favoring stable nodes, the algorithm adjusts *TTR* values depending on the permanency of the nodes in the overlay network (the longer the permanencies, the higher the values).

For the sake of clarity, we reproduce here some definitions in overlay terminology:

- Graceful and ungraceful leaving: A graceful peer departure from the overlay implies a notification to the overlay in which the peer transfers its routing and resource information. In an ungraceful departure (*i.e.*, due to link failure), the registered information on the lost peer stays in the overlay.
- Parallelism (*α*): the number of parallel messages that a peer can send to other peers to accelerate operations.
- Replication (*r*): the number of peers responsible for each contact or resource information.
- System-wide number (*k*): the number of entries in each list or bucket of peers composing the DHT.

The rest of this paper is organized as follows. Section 2 describes the background. Section 3 discusses related work. Section 4 presents our proposal for adaptive network updates. Sections 5 and 6 evaluate the proposal by means of analysis techniques and simulations, respectively. Finally, section 7 concludes the paper.

## 2. Background

### 2.1. SIP and Sensor Information Exchange

SIP was originally designed for VoIP and instant messaging applications. However, it is currently being used in applications beyond its original purpose, such as sensor information exchange. For example, SIP has been proposed as a protocol for metering information exchange in the Smart Grid [19,20], intelligent transport and mobility systems [21–23] and mobile service provision [24]. All these services rely on information provided by sensors.

Traditionally, wireless operators have largely ignored device-to-device (D2D) communications, because they considered that they were only useful to reduce the cost of service provision [25,26]. However, in recent years, this has changed, mainly for two reasons. Firstly, practical context-aware applications have emerged thanks to the many sensors available in current commercial terminals and their ubiquitous data connectivity. Since collaborative context-aware applications need to gather information from nearby devices, efficient D2D communications are necessary to discover these devices and exchange sensor information with them. Secondly, machine-to-machine (M2M) applications are growing fast. In this scenario, operators have already announced the development of the LTE-direct specification, for which P2PSIP may fulfill the requirements for information exchange between neighbor devices [27,28].

The popularization of mobile devices, such as smart phones, tablets and laptops, and the fast growth of wireless sensor networks, which are strongly power limited [29,30], have driven the development of energy-aware protocols. To avoid battery depletion, these protocols seek to minimize management message transmission. In this regard, P2PSIP is highly reliable and is also efficient in terms of power consumption [31].

### 2.2. P2P Overlays

P2P overlay networks are collections of nodes (called peers) interconnected by *logical* links (each logical link may comprise several physical links), conforming a distributed system without hierarchical organization or centralized control. Peers can join or leave the network at any time, meaning that typical overlay networks are dynamic and require some form of topology management.

The design space of P2P overlays is wide, and various classifications are possible. One of these divides P2P overlays into *structured* and *unstructured* overlays:

- Structured overlay. This involves key-based routing. The peers maintain routing tables and exchange these periodically for maintenance, *i.e.*, to support message delivery if there are no failures. Structured overlays are fully distributed and are usually characterized by a geometry, a routing algorithm and a maximum number of hops for routing a request. Since the information sought stays at specified locations, subsequent queries are efficient if the network is stable.
- Unstructured overlay. In this scenario, peers use flooding techniques to send queries across the overlay.

Flooding does not scale well. Moreover, some analytical studies on unstructured solutions often make topological assumptions that are incompatible with multi-hop wireless networks and ignore link vulnerability [32–34]. In structured overlay networks, the DHT mechanism allows self-organization with efficient routing, high search accuracy, high scalability and automatic load balancing [15]. We use the Kademlia DHT-based algorithm based on the XOR metric in our work. Nonetheless, our proposal can be extended to other structured overlays.

Structured overlay maintenance is costly, yet crucial, for P2P network performance. Most structured overlays use key-based routing, where each peer has a DHT to store a portion of the key-based routing information. DHT-based systems require *O* (log *N*) hops on average to locate any data in the overlay.

Data-centric P2P applications commonly use replication to ensure high availability, even if the nodes leave the network ungracefully (without transferring information to other nodes). The peers exchange management messages periodically to keep published information in the overlay up-to-date. Frequent refresh of relevant data in the overlay results in high overhead, due to management messages. However, an overlay on top of a highly mobile wireless network is unreliable if refresh actions are too widely spaced.

Energy efficiency is another important issue in peer-to-peer overlay networks. Zhang *et al.* [35] demonstrated that transmitting one bit in a wireless network requires 1,000 times the energy of a single 32-bit computation. Obviously, this means that management techniques should use as few messages as possible.

### 2.3. Kademlia

Kademlia assigns 160-bit cryptographic hash IDs to peers and provides a lookup algorithm. Its main advantages are scalability and *O* (log *n*) search complexity, *n* being the number of peers. Each peer maintains a DHT with information about other peers to be used for routing schemas, and each Kademlia message includes peer-ID information about the sender.

Table 1 summarizes some notations used in the definition of Kademlia.

Kademlia structures its ID space like a tree. The DHT is divided into *k*-buckets, where *k* is the number of entries in each bucket. A *k*-bucket is a list of *k* peers within a certain distance (in the XOR metric) of the current peer. For instance, the distance between 0001 and 0111 (four-bit IDs) in a XOR metric is six. This results in *kN* entries in the routing table.

Note that the XOR operation defines a non-Euclidean metric (*d*(*p_i_*,*p_j_*) = *p_i_* ⊕ *p_j_*) with the following properties:

(1) $$\begin{array}{cc} d(p_{i,}p_{i}) & =\;0\\ d(p_{i,}p_{j}) & >\;0,\forall p_{i}\neq p_{j}\\ d(p_{i,}p_{j}) & =\;d(p_{j,}p_{i}),\forall p_{i}\neq p_{j}\\ d(p_{i,}p_{j})+d(p_{j,}p_{k}) & \geq \;d(p_{i,}p_{k}) \end{array}$$

The XOR metric is unidirectional, which ensures that all lookups converge along the same path:

- Each *k*-bucket is sorted by last contact time (contact information, including peer-ID, IP address and port).
- When a message is received and the *k*-bucket is full, the receiver peer checks if the node in the *k*-bucket that was checked the last time is on-line: if there is no response, the node is removed, and the new peer is inserted at the tail. Otherwise, the new peer is discarded. Thus, Kademlia favors stable nodes.

The more contacts per bucket (achieved by increasing *k*), the more the DHT will be protected against churn. This is because peers are faster at repairing the routing table when a failure is detected. The DHT becomes more resistant to churn by accumulating high-quality contacts.

Kademlia uses strict parallel routing to accelerate registration and lookup operations according to the system-level concurrency parameter (*α*). Increasing *α* reduces latency, but the overhead increases roughly linearly as a result [36].

Kademlia employs iterative, recursive or semi-recursive algorithms for node lookups. A lookup consists of a sequence of lookup steps (or hops). In iterative routing, an intermediate node always replies to the originating peer, which will then send the message to the peer that is the next hop. In recursive routing, an intermediate node in a path will send the messages directly to the next peer. The response unwinds and follows the same path back. Semi-recursive routing is similar to recursive routing, but the response is sent directly to the requester. In all cases, the initiating peer sends *α* messages to the closest nodes (to the requested key).

To ensure the persistence of <key, value> pairs, registry peers republish the keys after the *TTR* timer expires. Registrar peers, on the other hand, handle *time to expiration* (TTE) timers, which are typically initialized to twice the initial value of the *TTR*, so that on TTE expiration, the corresponding registered peer data is considered stale and removed. There is a timestamp associated with each contact or resource. If the timestamp corresponding to a particular contact or resource item has not been updated when the peers check their *k*-buckets and resource tables, the item will be considered stale and be removed (it is considered to correspond to a peer that has left the overlay ungracefully).

### 2.4. The P2PSIP Protocol

P2PSIP implements traditional proxy and registrar SIP functions in a distributed way. Resource information is distributed among all the peers in the overlay, and requests are also handled by the overlay infrastructure. The main advantages of P2PSIP are cost reduction and the elimination of single failure points. Using P2PSIP, any DHT-based P2P network can locate resources (services or users) in a decentralized way.

A P2PSIP deployment has an overlay name, and the participants can be peers (active) or SIP clients (passive). The peers are identified by a peer-ID, and they collectively serve as a directory service for locating resources using the defined DHT structure. The clients only use resources from the overlay. They do not participate in its maintenance.

The information stored in the P2PSIP overlay consists of registers of the peer nodes and the resources available at these nodes. Kademlia's distributed resource tables (DRTs) are similar to DHTs employed for peer registration. The same procedures as those described in Section 2.3, based on unique resource-IDs and a XOR metric, are employed. In order to keep registers up-to-date, they are periodically refreshed. Section 3 discusses previous algorithms for managing refresh time, whereas Section 4 describes the novel algorithm in the present work.

Protocol dSIP [5] uses SIP messages to implement P2PSIP, while preserving the semantics of conventional SIP messages as much as possible. Although there are newer P2PSIP implementations, dSIP has several advantages:

- Simplicity of implementation (still text-based)
- Minimization of the number of protocols required for a P2P UA.
- Easy integration into existing UAs and reuse of available SIP stack implementations.
- Widespread support.

The message to add, remove and query bindings in DHT and resource tables is SIP REGISTER [37]. dSIP supports:

- Peer and resource registration.
- Session establishment.
- DHT maintenance (dSIP is modular, so it allows multiple DHTs).

dSIP peers are active members of the overlay and provide operations to enable self-organization (SIP server-like functions) in addition to the basic functionality of any SIP endpoint. The dSIP overlay serves as a directory service for locating resources.

## 3. Related Work

Li *et al.* compared the performance of the most relevant protocols for structured overlay networks under churn [38], including Kademlia, and concluded that they perform similarly with well-tuned periodical maintenance tasks. Maintenance of routing information stored in distributed DHTs is complex under churn, and it may saturate the network [39]. Although Li *et al.* focused on wired networks, their conclusions are also valid for wireless structured overlays. Cirani and Veltri defined a graceful leaving system, where peers issue unregistration messages for routing tables and resource registers [5]. In wireless networks, however, these messages may get lost, for instance, when a peer leaves the coverage area or loses connectivity due to terrain obstacles.

Successful implementations of structured overlays using Kademlia can be found in [40-43]. *K*-Umbrella [44] is a DHT algorithm that extends Kademlia using *k*-ary rather than binary trees.

Rhea *et al.* demonstrated by simulation that periodic recovery is more efficient than reactive recovery when a large overlay has reasonable churn rates [45]. Ou *et al.* evaluated Kademlia in mobile environments with different degrees of churn [8], setting adequate fixed refresh periods. They concluded that a choice of *k* = 3, *α* = 3 and *r* = 3 makes the overlay resilient to ungraceful leavings. In [46], Koskela *et al.* proposed community overlays, an approach based on multiple overlapping overlays, and showed that they performed better. However, none of these analyses considered messaging load. On that topic, Maenpaa and Camarillo studied P2PSIP overlay network management in terms of signaling bandwidth, lookup delay and failed lookups for fixed refresh periods [47]. Chan *et al.* also measured bandwidth consumption for the same scenario [48]. Refresh periods for DHT algorithms were necessarily small for wireless networks under churn, implying significant signaling. Kelenyi and Nurminen reduced management traffic by means of probabilistic incoming message dropping [49], but this strategy affects routing performance.

Finally, some techniques employ a dynamic *TTR*. The schema in [50] follows a simple algorithm with additive increase and multiplicative decrease (*AIMD*). At each period, the algorithm defines a new *TTR* value based on the result of the previous updates. If resource locations do not change, the overlay is assumed to be stable, and the *TTR* can be incremented; otherwise, it must be decremented:

(2) $$\text{TTR}=\{\begin{array}{ll} {\text{TTR}}_{\text{old}}+C & \left(\text{nocongestion}\right)\\ {\text{TTR}}_{\text{old}}\times D & \left(\text{othrewise}\right) \end{array}$$

where *C*, *C* > 0 and *D*, 0 < *D* < 1 are constants. In addition, *AIMD* sets higher and lower limits for the new *TTR* values.

In the alternative approach in [51], refresh timers vary linearly. This approach therefore adapts poorly to network changes compared to *AIMD* in terms of the probability of stale information in the overlay. Accordingly, we selected *AIMD* to compete with our own dynamic proposal in Section 4.

Summing up, reducing the maintenance load of overlay networks over wireless networks is a challenging problem. The most advanced algorithms in the literature reduce this cost by dynamically adapting maintenance intervals (with dynamic refresh times). In this work, we present better *TTR* adaptation techniques with the same goal.

## 4. The ATTR Schema

Our schema seeks to provide adaptive *TTR* values to minimize management signaling load while keeping a low probability of unreliable information, which can degrade routing performance. As described in Section 2, Kademlia usually defines a fixed *TTR* value, although a dynamic value is technically possible.

In the original Kademlia implementation, and in the proposals in the literature, each peer has a single refresh timer. When the timer expires, the peer re-registers its information in the overlay.

In this work, we refer to a Kademlia overlay with static *TTR* values as the **original schema.**

Our *ATTR* solution involves different timers for each registered resource to populate peer information across the overlay (to update routing tables). Within the SIP REGISTER message, each peer sends a timestamp that the receiver uses to compute the corresponding latency. The response to this SIP REGISTER message includes that latency estimation, so that the sender can define the new timeout for the corresponding resource. The use of custom timers for each resource reduces message bursts considerably in comparison to the other proposals in our analysis.

As each peer in the overlay is responsible for a part of the DHT identifier space, peer churn causes two types of inaccuracies in routing and resource tables:

- Peers that still lack contact and resource information about a recently arrived peer.
- Peers with stale contact and resource information about recently departed peers.

The *TTR* calculation problem focuses on the second type of inaccuracy, because arriving peers push contact and resource information upon arrival. Our proposal, which we will call the *ATTR schema*, outperforms the adaptive method of reference, *AIMD*, by taking into account the latencies between the registered peer and the registration destination. This is achieved in two ways: low latencies are taken into account to adjust the *TTR* additive increase, and the new *TTR* is reduced when latencies exceed their average, due to expected peer losses, enabling earlier detection of peer outages.

The adaptive *TTR* (*ATTR*) values are calculated at the peers that receive the SIP REGISTER message (hereafter, *registrars*). The refresh procedure of each sender peer or resource (hereafter, *registry*) sets its associated timer accordingly. This approach allows refresh times to evolve independently. The registrar computes the *ATTR* values and inserts them in the “SIP 200 (OK)” message (received message is correct) in the *Contact* field (as the dSIP implementation does with other attribute-value pairs, e.g., peer-ID). All SIP messages modified by the *ATTR* schema are SIP-compliant. Peer and resource information is not replicated at the same peers in the overlay (e.g., peer information can be registered at peers *i*, *j* and *k*, and resource information can be registered at peers *x*, *w* and *z*).

The *ATTR* schema defines the following parameters:

- Initial refresh time (*T_init_*). Initial refresh time value.
- Permanency time (*T_perm_*). Time from the moment the registry joined the overlay. When a peer leaves the overlay, this parameter is reset to zero.
- Registration latency (*Lat_reg_*). Time from the moment a registry sends a SIP REGISTER message to the point at which the registrar receives the request.
- Mean registration latency (*Lat_mean_reg_*). Average latency for each registry based on the registration latency computed by the registrars in the overlay for each registration message they receive.

Because the refresh time is calculated at the registrars (local refresh time), the confirmation response must contain the *ATTR* to be used by the registries. This way, information about the same peers or resources is registered in the overlay with different expiration times, depending on the latency between peers.

In a multi-hop network, delays between source and destination can be considerable and depend on the number of hops and the link delays. A sudden increase in delay may mean that a link will fail soon. The refresh time calculated in these conditions will therefore be short (the reduction will depend on the ratio between the current delay and the average delay).

Specific *ATTR* values are calculated from the *Lat_mean_reg_* historical record and the time that the registrar obtains from the SIP REGISTER messages. For this purpose, the registry adds a timestamp to the original SIP message (the same procedure as for the *ATTR* value in the SIP 200 message), and the registrar obtains the latency by comparing the timestamp with its current time (the registrar is aware of the registry identifier or peer-ID). Thus, to achieve correct latency measurements, the overlay must be synchronized (either with a distributed network time protocol or with GPS, which is widely available in current mobile terminals). The registrar also records the registry (or resource) in a table with the expiration time *TTE* = 2 × *ATTR*. Later, as previously mentioned, the registrar answers the SIP REGISTER message with an SIP 200 (OK) message (which we have also modified) that includes the *ATTR* value it calculated.

Peer and resource information is registered in the overlay according to a replication parameter (*r*), such that a registry sends *r* SIP REGISTER messages to the overlay and receives *r* SIP 200 (OK) messages from the registrars. The registry executes Algorithm 1 to update its *TTR* initial values with the maximum *ATTR* value received. This ensures that peer or resource information will not be in the overlay when the associated timer expires.

The schema computes *ATTR* values as:

(3) $${\text{ATTR}}_{j}^{i}=\max\left(T_{\text{init}}+\frac{\log(T_{\text{perm}}^{i})}{\log(1+1/T_{\text{init}})}-F_{\text{lat},j}^{i},T_{\text{init}}\right)$$

where 0< *i* ≤ *N_peers_* and 1< *j* ≤ *N_resources_* + 1. The main elements of the formula are:

- Maximization *versus T_init_*, so *ATTR* ≥ *T_init_*. Thus, the overhead of the *ATTR* schema is upper-bounded by that of an original schema with (a fixed) *TTR = T_init_*.
- The additive component log( $T_{\text{perm}}^{i}$)/log(1 + 1/*T_init_*) determines an *ATTR* logarithmic growth step when the nodes do not experience any trouble. By contrast, *AIMD* applies a constant growth step. Hence, registries that stay in the overlay for long will have lower *TTR* values under the *AIMD* schema, yielding excessive overhead.
$-F_{\text{lat},j}^{i}$ represents the influence of delay on refresh time calculation. This subtractive component plays a role similar to that played by multiplicative decrements in *AIMD* under congestion. However, the *ATTR* schema is responsive to latency excess and not to congestion. In other words, ATTR values decrease only when the target peer is unusually hard to reach (an outage is expected) and not when it is subject to congestion.

**Algorithm 1:** Peer/resource registration update in the overlay.
 *r*, replication parameter, *α*, concurrency parameter *n*, number of SIP REGISTER messages sent *timer_j_*, the refresh timer for resource*_j_* or peer information, is set to *T_init_* initially **if**
*timer_j_* expires **then**  select resource*_j_* or peer information  set *n* = 0  **while**
*n* < *r*
**do**   **if**
*α* > (*r* ‒ *n)*
**then**    *x* = *α*   **else**    *x*= *r–n*   **end if**   select the *x* closest peers to resource-ID*_j_* or peer-ID (not previously selected) from the DHT   send *x* SIP REGISTER messages (resource*_j_* or peer information)   *n* = *n*+*x*   **while**
*x* > 0 **do**    wait for SIP 200 (OK) or SIP REGISTER timeout    decrement *x*    **if** SIP 200 (OK) **then**     *timer_aux* ← *get ATTR from message*(SIP 200 (OK))     **if***timer_j_* < *timer_aux***then**      *timer_j_* = *timer_aux*     **end if**    **end if**   **end while**  **end while**  **if***timer_j_* ≤ *T_init_***then**   *timer_j_* = *T_init_*   Re-register the remaining resources and peer information   (if *timer_k_* > *T_init_*) with a defined *TTR* value (*T_init_*).  **end if** **end if**


The latency excess response parameter is computed as:

(4) $$F_{\text{lat},j}^{i}=\{\begin{array}{ll} \exp\left(\frac{2Lat_{\text{reg},j}^{i}}{Lat_{\text{mean}\_\text{reg},j}^{i}}\right) & \text{if}Lat_{\text{reg},j}^{i}>Lat_{\text{mean}\_\text{reg},j}^{i}\\ F_{\text{lat},j}^{i}\cdot \text{tune} & \text{otherwise},\text{with}\;0<\text{tune}<1 \end{array}$$

where the first part is exponential to respond rapidly to above-average latencies and the second part is linearly responsive to below-average latencies. This second term is multiplied by a *tune* parameter to configure the speed of recovery from spurious delay increments. The *ATTR* schema follows the philosophy of Kademlia in the sense that it depends on permanency time, because the oldest peers are most likely to stay alive [16]. It is also parametric and depends on the initialization parameter, *T_init_*, and the recovery parameter, *tune*. The excess latency response factor, *F_lat_*, allows the schema to adapt to network conditions. As there is a different timer for each resource, as well as for each peer, there are fewer management message bursts than in the original schema. When a *TTR* timer expires, the associated peer and resource data are re-registered. The corresponding registration messages contain timestamps, and the registrar responses contain the *ATTR* values calculated with Equation (3). The registry will choose the largest of these values. This ensures that, given *r* registrars, the TTE timer of the best registrar (with the smallest latency) will not expire, even though the other timers may. This way, registration refresh is achieved at the minimal overhead cost.

When all the updating values received lie below threshold *T_init_*, all other resource and peer information is re-registered with that minimum *ATTR* value, since a timer updating value below the threshold may indicate that a registry is likely to leave the overlay. This prevents stale information from remaining in the overlay for too long. Registries flush their registrations preventively when they find themselves likely to fail.

When remote registrars send low update values in their SIP 200 (OK) messages with low *ATTR* values, they are ignored by the registry, which instead chooses registrars with higher update values, because they are more likely to stay in the overlay (the probability of ungraceful departure is low). When all the *ATTR* values received are low, the probability of ungraceful departure is high. If the selected (highest) *ATTR* is below the threshold, the registry will automatically re-register itself (peer-ID) and all its resources with an associated *ATTR* that exceeds the threshold. This special registration uses SIP REGISTER messages with the defined minimum refresh time value, *T_init_*.

## 5. Analysis

To evaluate message overhead due to overlay maintenance in the original schema (periodic refresh times), we first assume that there is no churn. Let us consider that each peer is responsible for *N_resources_* resources and uses iterative lookup. According to [5], for a periodic refresh time, the average number of management messages per minute is:

$$N_{\text{messages}}\leq \left[2\cdot (k\cdot (N_{\text{resources}}+1)+(\log_{b}(N_{\text{peers}}-1)+c)\cdot N_{\text{resources}})\right]\text{ratio}$$

where *ratio* is defined as the number of refresh periods per minute. Equivalently, *ratio* = 1/*TTR*. Therefore, both *AIMD* and *ATTR* need fewer management messages, because they allow increases in *TTR*. Each peer sends *N_resources_* +1 registration messages (representing itself and its resources) to the *r* closest peers. It also receives the same number of registration messages from the peers in the overlay, so it has to answer them. The iterative lookup to find suitable peers for *N_resources_* + 1 registrations requires up to *log_b_*(*N_peers_* – 1) + *c* messages, where *c* is a small constant.

**Figure 1.**
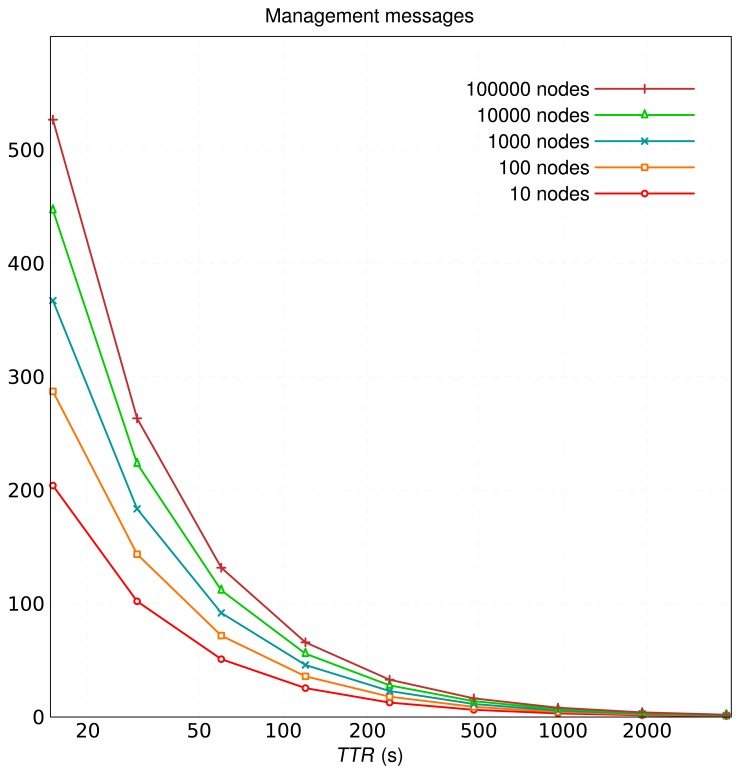
Number of management messages sent per minute for different refresh periods and population sizes, original schema.

Figure 1 shows the average number of management messages per minute in the original schema for different population sizes (10, 100, 1,000, 10,000 and 100,000 peers in the overlay), with *r* = 3. The number of peers has less impact on the number of management messages than the replication factor. Signaling load is unacceptable for short refresh times.

However, if there is churn, for example, if the peers belong to an unstable wireless network, they will leave ungracefully with a certain probability, *p_term_* (defined for a one-minute time frame). Peer departure from the overlay is not notified, and the average time between departure and stale information expiration is a function of the remaining *TTR* and the expiration timer. The remaining *TTR* is:

(5) $$\bar{TTE_{\text{rem}}}=\int_{0}^{\text{TTR}}tf_{\text{term}}(t)dt$$

where *f_term_*(*t*) is the distribution of ungraceful departures during the *TTR* cycle. Assuming a uniform distribution:

(6) $$\bar{TTE_{\text{rem}}}=\frac{\text{TTR}}{2}$$

The expiration timer is originally set to *TTE* = 2 × *TTR*, so at the moment of departure, the time remaining to resource expiration is:

(7) $$\bar{TTE_{\text{rem}}}=\text{TTE}-(\text{TTR}-\bar{TTE_{\text{rem}}})=\frac{3}{2}\text{TTR}$$

Meanwhile, the stale information will remain registered in the overlay.

In addition to reducing management load, adaptive refresh times also decrease the probability of stale information due to ungraceful departures. The *ATTR* schema updates the refresh time with the highest value from the *r* SIP 200 (OK) messages received. If the registry is far from the registrars, the delay measured using SIP REGISTER arrival times and timestamps will be long. *F_lat_* will also be high, and the refresh time will be short. In addition to reducing the probability of stale information, the time before stale registered information expires is parametric in *ATTR*, and it can be computed using Equation (7), assuming that the latency-based departure anticipation mechanism succeeds in making *ATTR* = *T_init_* iust before the event:

(8) $$\bar{TTR_{\text{proposal}}}=\frac{3}{2}T_{\text{init}}$$

Therefore, thanks to a detection procedure for unannounced departures, stale information about peers that have left ungracefully only remains in the overlay for a short time, adjustable by the initial refresh time. As the peers do not refresh all the management data periodically, the probability of management information exchange between peers during ungraceful departure increases and the time that stale information remains in the overlay will tend towards the ideal limit defined in Equation (8).

In contrast to our proposal, the *AIMD* schema does not consider delays between peers (it only employs congestion estimation) and, therefore, does not anticipate ungraceful departures. Moreover, the *AIMD* schema continuously performs refresh procedures for all resources at the same time. This means that management messages travel in bursts. The *ATTR* schema only behaves in this way when an ungraceful departure is predicted from a latency excess. In addition, congestion is estimated less frequently in *AIMD* than latency in the *ATTR* schema, increasing the chance that in the *AIMD* system, departures will occur during higher values of $\bar{\text{TTE}}$ than in the *ATTR* schema. Therefore, with the *AIMD* schema, stale information remains in the overlay for longer than with the *ATTR* schema.

## 6. Simulation

### 6.1. Simulation Set-up and Merit Metrics

The OMNeT++ network simulator [52] was employed for the tests. The scenario we considered was a 100 × 100 m^2^ square area with different peer population sizes, ranging from four to 100 randomly placed nodes. All the peers were mobile, with speeds of between eight and 20 m/s, following a Gaussian distribution. The underlying wireless *ad hoc* network was an IEEE 802.11s mesh. The simulations were repeated with 100 different random seeds for different population sizes. Each simulation lasted 3.600 s.

**Figure 2.**
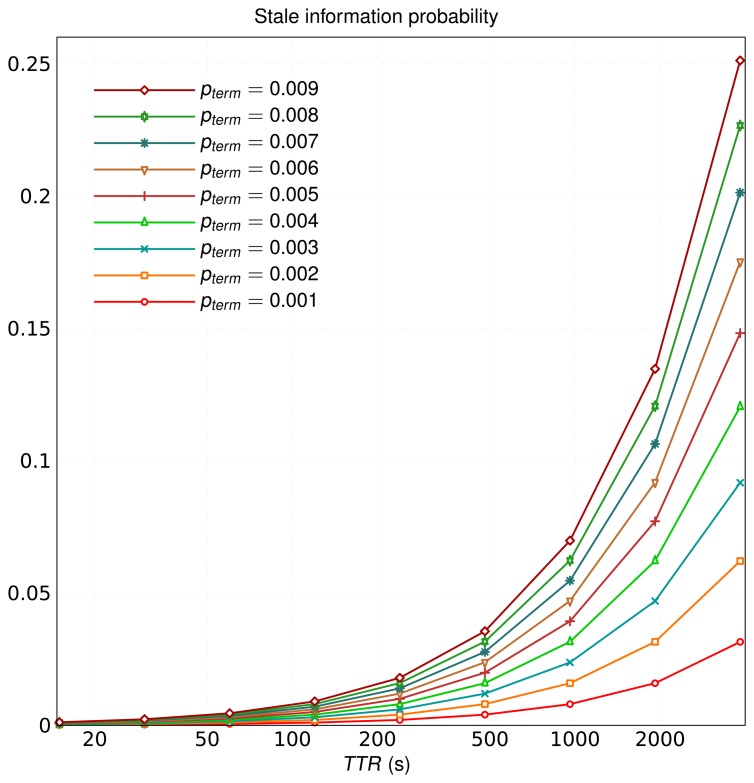
Probability of stale information in the overlay with the original schema.

We simulated the original schema, *AIMD* [50] and our proposal to determine which had the lowest overhead of management messages for a comparable probability of stale information. We must emphasize that the logical links between overlay nodes were the same for the three schemas, since we did not notify the routing management in Kademlia. DHT information was always registered in the same nodes regardless of the schema we used. It is important to recall that, in the original schema, the probability of stale information in the overlay increases with *TTR*, as shown in Figure 2, according to:

(9) $$p_{\text{stale}\_\text{information}}=1-{\left(1-p_{\text{term}}\right)}^{\bar{TTR_{\text{rem}}}/60}$$

where *p_term_* is the probability of node departures occurring in one minute, and thus, *p_stale_information_* is the joint probability of this event within a $\bar{TTE_{\text{rem}}}/60$ min window.

The management messages considered were SIP REGISTER and SIP 200 (OK) messages from the dSIP protocol. The DHTs and resource tables were fed by processing those messages. The lookup procedure also used dSIP register messages.

Moreover, the following assumptions were made:

- DHTs used iterative routing.
- Peers could leave or join the overlay (so there was churn).
- Peer identifiers were not uniformly distributed, and therefore, the number of entries in each *k*-bucket differed.

We adjusted the parameters of the original schema (*TTR*), *AIMD* (*T_init_*, *C* and *D*) and *ATTR* (*T_init_*, *tune*) for the same probability of stale information. This was done by inspection. The initial *TTR* was 15s for the original schema, so the peers performed four refresh actions per minute. *T_init_* was 15s for the *AIMD* and *ATTR* schemas, and thus, the mean permanency time of stale information with *AIMD* was the same as with our proposal. The replication and concurrency factors were three. Each peer registered three resources in the overlay. For *AIMD C* and *D*, we chose values that would allow moderate capacity variations, in order to achieve a probability of stale information comparable to that of *ATTR*. High *C* and *D* values would make it difficult for *AIMD* to determine that a node has left the overlay. Finally, the *tune* parameter was set to 0.875 for *ATTR* to keep the same probability of stale information as with the original schema. Availability was practically the same in the three scenarios. The examples in Section 6.2 correspond to *p_stale_information_* = 10^–3^.

### 6.2. Comparison

**Figure 3.**
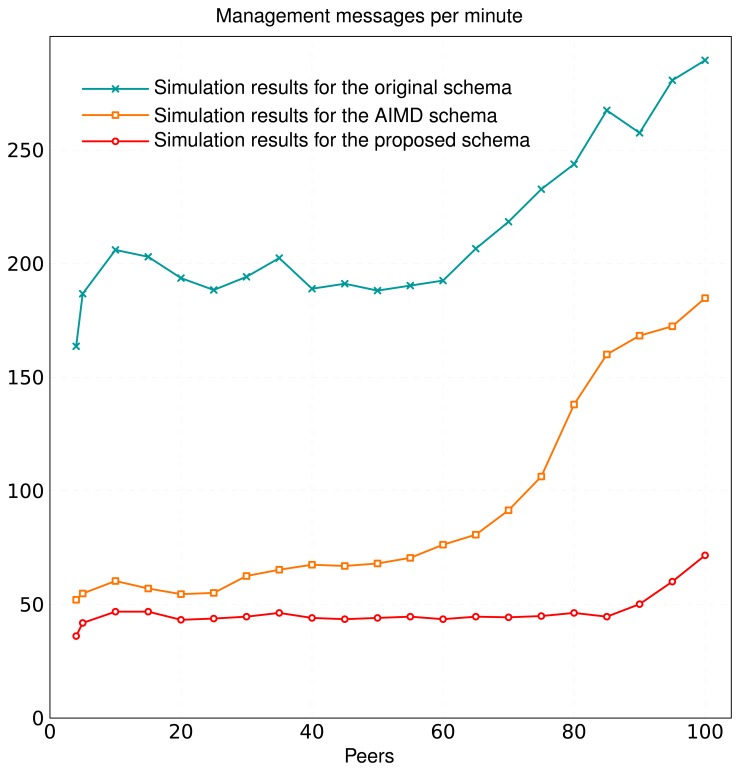
Comparison of management overhead for different population sizes (from 4 to 100 peers). Initial refresh time of 15 s for all schemas (*p* = 0.001.).

Figure 3 compares the results of the schemas analyzed, for a confidence interval of 95% and a tolerance interval of 1%. The average number of management messages per minute with *ATTR* was much lower than with the original schema and *AIMD*, even for overlays with a few nodes. This difference became more significant as the number of nodes grew.

The *ATTR* schema achieved considerable improvements in terms of management overhead (approximately 21.50% of the number of maintenance messages in the original schema). Furthermore, *ATTR* required half the management messages of *AIMD*. We can illustrate this with an example. The average size of a management message in the Kademlia + dSIP implementation by the University of Parma is 689 bytes. Other P2PSIP implementations, such as RELOAD [53], have even larger average management message sizes. Using the original schema with a 100-node overlay, the management traffic rate is 26.182 kbps. With the *AIMD* schema, this rate is 16.995 kbps, and with our schema, it is only 10.565 kbps.

In addition to the results in Figure 3, the simulations also checked *p_stale_information_*. We verified that its value was ∼10^–3^ for the three schemas, so they were correctly tuned. Indeed, the probability of stale information was lower in all cases with *ATTR* than with *AIMD*. The information about the peers that left the overlay ungracefully persisted only for a short time (upper-bounded by the *time-to-refresh* of the original schema).

## 7. Conclusions

Wireless networks are evolving towards all-IP solutions that will rely on SIP for management purposes and sensor information exchange. In these networks, SIP technologies may follow a P2P approach, to enhance availability, storage and processing resources and scalability. In this context, P2PSIP protocols allow decentralized and fast communications. However, P2P mobile sensing overlays on top of wireless networks require further improvements in terms of management overhead and battery lifetime, which are closely related.

In this paper, we have presented a novel approach for reducing the traffic of management messages needed to maintain mobile sensing overlays on top of wireless networks, considering a Kademlia + dSIP P2PSIP implementation.

In the original Kademlia schema, based on periodic maintenance, short maintenance periods have a clear negative impact on overlay performance, since they increase management overhead. Furthermore, long maintenance periods increase the probability of stale information, which can lead to routing failures or longer lookup times due to timeouts.

We have proposed a new adaptive *TTR* (*ATTR*) schema that reduces management traffic while keeping the probability of stale information in the overlay at acceptable levels. Our simulations show that, for the same probability of stale information, our proposal outperforms both the original schema with fixed periodic refresh times and *AIMD*, one of the best dynamic *TTR* alternatives so far.

Our solution reduces management traffic and, as a consequence, it also mitigates link overload and bottlenecks. In forthcoming work, we will evaluate overload decrease in real mobile mapping and routing (MMR) networks, in which peers are aware of the topology. This configuration will allow the creation of logical links according to hop counts or delay measures and achieve faster lookups and, therefore, improved performance.

## Figures and Tables

**Table 1.** Variable and function definitions.

**Symbol**	**Meaning**
Peer-ID	Univocal peer identifier in the overlay.
Resource-ID	Univocal resource (user or service) identifier in the overlay.
*N*	Size (in bits) of the peer-ID or resource-ID (Kademlia uses *N* = 160, the length of an SHA-1 digest).
*S*	160-bit identifiers for the space address.
*d*	Distance function.
*p_i_*	Identifier of peer *i*.
⊕	Exclusive OR.
*N_peers_*	Number of peers in the overlay.
*N_messages_*	Number of management messages sent.
*N_resources_*	Number of resources that each peer registers in the overlay.
